# Multiple Myeloma from the Perspective of Pro- and Anti-Oxidative Parameters: Potential for Diagnostic and/or Follow-Up Purposes?

**DOI:** 10.3390/jpm14030221

**Published:** 2024-02-20

**Authors:** Ayse Nilgun Kul, Bahar Ozturk Kurt

**Affiliations:** 1 Department of Hematology, Kartal Dr. Lütfi Kırdar City Hospital, Istanbul 34865, Turkey; 2 Department of Biophysics, Cerrahpasa Medical Faculty, Istanbul University-Cerrahpaşa, Istanbul 34098, Turkey; bhroztrk@iuc.edu.tr

**Keywords:** multiple myeloma, oxidative stress, antioxidants, biomarkers, malondialdehyde, nitric oxide

## Abstract

Background: Multiple myeloma is a hematological malignancy characterized by anemia, antibodies causing kidney damage, and damage to multiple organs, which come together to cause morbidity. Although oxidative stress is not a core pathological aspect of multiple myeloma, reactive oxygen species (ROS) and antioxidant balance have been shown to play a role in the disease process and are considered in its management. In the presented study, we aim to assess the reliability of specific oxidant and antioxidant variables as potential biomarkers for multiple myeloma and to determine which of these variables might exhibit higher sensitivity in predicting multiple myeloma. Methods: This case-control study was conducted between March 2023 and August 2023. A total of 30 multiple myeloma patients, newly diagnosed according to the multiple myeloma diagnostic criteria revised by the International Myeloma Study Group in 2014, and a total of 30 volunteers without multiple myeloma were included in this study. Serum glutathione peroxidase (GSH-Px), superoxide dismutase (SOD) and catalase (CAT), and malondialdehyde (MDA) and nitric oxide (NO) levels were measured with the first blood samples taken after inclusion. Results: The groups had similar age (*p* = 0.623) and sex distribution (*p* = 1.000). MDA (cut-off: >4.35, *p* < 0.001), GSH-Px (<59.8, *p* < 0.001), CAT (<67.2, *p* < 0.001), SOD (<21.2, *p* = 0.001), and NO (>38.5, *p* < 0.001) could significantly detect multiple myeloma. Multivariate logistic regression revealed that increased MDA (*p* = 0.003) and NO (*p* = 0.001) levels and decreased GSH-Px (*p* = 0.001), CAT (*p* = 0.001), and SOD levels were independently associated with multiple myeloma disease. Conclusions: The presence of increased antioxidant levels and decreased antioxidant levels in patients with multiple myeloma is the clearest indicator of increased oxidative stress. These parameters may help to identify potential therapeutic targets and develop strategies to control disease progression.

## 1. Introduction

Multiple myeloma is a plasma cell dyscrasia characterized by the accumulation of clonal cells in the bone marrow and overproduction of monoclonal immunoglobulins, presenting with hypercalcemia, renal failure, anemia, and bone lesions [1]. It accounts for 1% of all malignancies and about 10% of all hematologic cancers, and the disease leads to considerable morbidity [2,3,4]. Plasma cells tend to generate high quantities of immunoglobulins, resulting in endoplasmic reticulum stress, an imbalance of redox homeostasis, and intracellular reactive oxygen species (ROS) production [1]. Although oxidative stress is not a core pathology of multiple myeloma, ROS and antioxidant balance have been demonstrated to play a role in the disease process and are considered in its management [5,6].

Although ROS modulates a number of metabolic actions at physiological concentrations, their excess production or deficiencies in the disposal can contribute to various adverse effects in many diseases [7,8,9] and genetic alterations [10,11,12]. Enzymatic antioxidant mechanisms such as catalase [13], glutathione peroxidase (GSH-Px) and superoxide dismutase (SOD) enzymes, and non-enzymatic antioxidants (glutathione, vitamins C, E, and A, and bilirubin) counteract the adverse effects of ROS [14,15,16]. Oxidative stress has been implicated in the development and progression of many diseases and malignancies, including multiple myeloma [1,17,18,19].

Reports indicate that antioxidants such as SOD, GSH-Px, catalase (CAT), and vitamins C and E are lower in patients with multiple myeloma, while serum levels of oxidative stress markers such as malondialdehyde (MDA) and advanced oxidation protein products (AOPP) are higher compared to healthy individuals [20,21,22,23]. Nonetheless, the oxidative balance in multiple myeloma is complex and multifaceted. There is no clear comprehension of the impact of elevated oxidative stress on multiple myeloma pathophysiology or myelogenesis among patients with multiple myeloma [5]. This complexity is best exemplified by the fact that some researchers have argued that inducing ROS may provide benefits in multiple myeloma due to cytotoxicity towards cells with high antibody production [5]. On the other hand, existing studies have shown an oxidant-to-antioxidant balance favoring oxidation, but most of these studies are relatively old publications [23,24,25,26,27]. In fact, there are various limitations in available research focusing on this topic. Some lack control groups [24], some have very limited patient counts [23,24,25], most do not report statistical analyses beyond basic statistics or descriptive data [21,23,24], and the majority lack sensitivity and specificity analyses for predictive performance. Therefore, we do not have sufficient data regarding the utility of certain oxidant or antioxidant parameters and their potential for use in the diagnosis or management of patients with multiple myeloma.

For all these reasons, we aimed to investigate whether various oxidant and antioxidant variables, including MDA, NO, GSH-Px, CAT, and SOD levels, can be used as reliable biomarkers for multiple myeloma and to assess which variables might be more sensitive in predicting multiple myeloma.

## 2. Materials and Methods

### 2.1. Ethical Considerations

This study was performed in line with the principles of the Declaration of Helsinki. Approval was granted by the local ethics committee (Date: 29 March 2023/No: #2023/514/246/6). All study procedures were explained to the participants, after which written informed consent was obtained from all.

### 2.2. Setting and Participants

This prospective case-control study was carried out in the Department of Hematology of Kartal Dr. Lütfi Kırdar City Hospital, Istanbul, Turkey, between March 2023 and August 2023. The patient group of this study comprised multiple myeloma patients who were newly diagnosed according to the multiple myeloma diagnostic criteria revised by the International Myeloma Study Group (IMWG) in 2014. All patients had been admitted to the hematology outpatient clinic of our hospital and were diagnosed within the aforementioned period. The control group of this study consisted of age-, sex-, and comorbidity-matched individuals who applied to the internal medicine outpatient clinic for various reasons other than multiple myeloma—given that they did not have any characteristics necessitating exclusion from this study. Individuals younger than 18 and older than 85 years of age, participants with a history or diagnosis of any malignancy (except multiple myeloma for the patient group), recipients of chemotherapy, those with chronic gastrointestinal disease, and those with active infection at the time of blood collection were excluded. In addition, subjects who refused to take part in research and patients with the following characteristics were not assessed for inclusion: individuals using antioxidant supplements within the prior 6 months (before the date of admission), smokers, and alcohol users.

### 2.3. Data Gathering and Laboratory Analysis

Participants were first questioned about their detailed medical history for eligibility. After inclusion, demographics and comorbidity data (diabetes mellitus, hypertension, ischemic heart disease, chronic obstructive pulmonary disease, and hypothyroidism) were recorded.

All laboratory measurements were performed in the Istanbul University-Cerrahpasa, Cerrahpasa Medical Faculty, using calibrated standard measuring devices and according to the manufacturer’s recommendations. The laboratory parameters included in this study were analyzed with the collection of an extra 5-mL blood sample (serum separator tube) from each subject, which was withdrawn as part of the routine process, and no additional invasive procedure was performed. Blood samples used for analyses were drawn before any treatment was started. The tubes were kept at room temperature for about 30 min to allow clot formation and then were centrifuged at 2000× *g* for 10 min. The serum SOD, CAT, and GSH-Px levels were measured in units of ng/mL according to the manufacturer’s manual using the Sunred Human SOD ELISA kit (SunRed Biotechnology Company, Cat. No: 201-12-0919, Shanghai, China) Sunred Human CAT ELISA kit (Cat. No: 201-12-5456), and Sunred Human GSH-Px ELISA kit (Cat. No: 201-12-0726), respectively. The serum MDA levels were determined using the Sunred Human MDA ELISA kit (Cat. No: 201-12-5380), and the NO levels were determined using the Sunred Human NO ELISA kit (Cat. No: 201-12-1511). MDA level was recorded in units of nmol/mL, while NO was recorded in μmol/L. All results were obtained via endpoint measurement at a wavelength of 450 nm with the BioTek-800TS absorbance reader (Agilent, Santa Clara, CA, USA).

### 2.4. Outcomes

The primary outcome of this study was to assess the differences between patients with and without multiple myeloma in terms of oxidant and antioxidant indicators. The secondary outcomes were to investigate which of these variables could be independently predictive for multiple myeloma and to compare the variables for their classification potential.

### 2.5. Statistical Analysis

All analyses were performed using IBM SPSS Statistics for Windows, Version 25.0 (IBM Corp., Armonk, NY, USA). For the normality check, the Shapiro-Wilk test was used. Data are given as mean ± standard deviation or median (1st quartile–3rd quartile) for continuous variables according to the normality of distribution (normal distribution vs. non-normal distribution, respectively). Frequency (percentage) values were described for categorical variables. Between-group analysis of normally distributed continuous variables was performed with a student’s *t*-test. Between-group analysis of non-normally distributed continuous variables was performed with the Mann–Whitney U test. Between-group analysis of categorical variables was performed with the chi-square test or Fisher’s exact test. The multiple myeloma prediction performance of the antioxidants was assessed using Receiver Operating Characteristic [28] curve analysis. Optimal cut-off values for discrimination were determined using the Youden index. Measurements of performance (sensitivity, specificity, accuracy, positive predictive value, and negative predictive value) were calculated based on the cut-off value. Logistic regression was performed to evaluate associations between antioxidants and multiple myeloma, with adjustment for age, sex, and comorbidity.

## 3. Results

A total of 30 multiple myeloma patients were included in the patient group, and a total of 30 volunteers were included in the control group. In this study, 56.67% (*n* = 17) of the patient group was male, and the mean age was 65.13 ± 8.80 years. Further, 60.00% (*n* = 18) of the control group was male, and the mean age was 63.87 ± 10.93 years. The groups had similar age (*p* = 0.623) and sex distribution (*p* = 1.000). Additionally, there were no significant differences for any of the comorbidities examined (all, *p* > 0.05). The patient group had significantly higher MDA (*p* < 0.001) and NO (*p* < 0.001) levels and significantly lower GSH-Px (*p* < 0.001), CAT (*p* < 0.001), and SOD (*p* = 0.001) levels compared to controls (Table 1).

**Figure 1:**
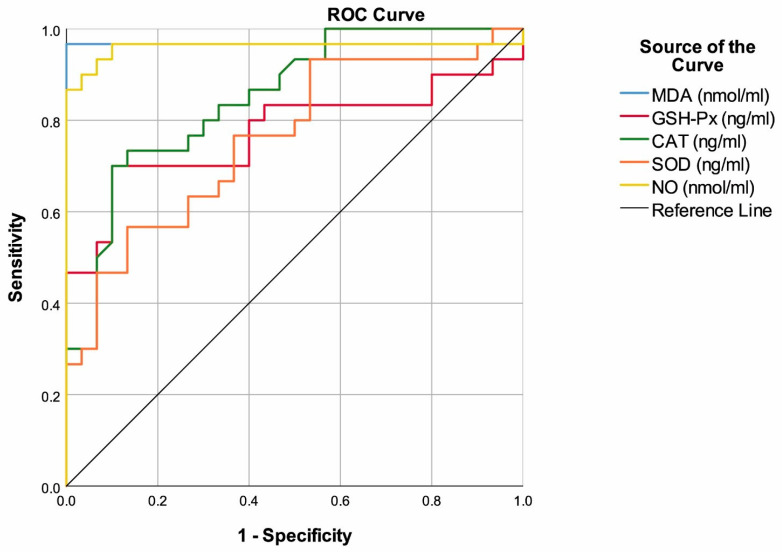
ROC curves of antioxidants to predict multiple myeloma.

MDA with a cut-off value of >4.35 had 96.67% sensitivity and 100.00% specificity to predict multiple myeloma. Additionally, the highest area under the ROC curve was found for MDA (AUC: 0.967, 95% CI: 0.902–1.000, *p* < 0.001). GSH-Px with a cut-off value of <59.8 had 70.00% sensitivity and 90.00% specificity to predict multiple myeloma (AUC: 0.773, 95% CI: 0.645–0.901, *p* < 0.001). CAT with a cut-off value of <67.2 had 73.33% sensitivity and 86.67% specificity to predict multiple myeloma (AUC: 0.850, 95% CI: 0.755–0.945, *p* < 0.001). SOD with a cut-off value of <21.2 had 56.67% sensitivity and 86.67% specificity to predict multiple myeloma but also the lowest area under the ROC curve (AUC: 0.760, 95% CI: 0.639–0.881, *p* = 0.001). NO with a cut-off value of >38.5 had 96.67% sensitivity and 90.00% specificity to predict multiple myeloma (AUC: 0.960, 95% CI: 0.895–1.000, *p* < 0.001) (Table 2, Figure 1).

Multivariable logistic regression (adjusted for age, sex, and comorbidities) revealed that high MDA (OR: 6.336, 95% CI: 1.886–21.289, *p* = 0.003), high NO (OR: 1.217, 95% CI: 1.084–1.366, *p* = 0.001), low GSH-Px (OR: 0.931, 95% CI: 0.892–0.972, *p* = 0.001), low CAT (OR: 0.910, 95% CI: 0.865–0.957, *p* < 0.001), and low SOD (OR: 0.895, 95% CI: 0.833–0.962, *p* = 0.003) were independently associated with multiple myeloma (Table 3).

## 4. Discussion

Over the past two decades, outcomes for multiple myeloma patients have greatly improved with new drugs and therapeutic approaches. However, most multiple myeloma patients are refractory to one or more treatment regimens, and some may also relapse after demonstrating a response to treatment. The disease is, therefore, still considered a serious cause of mortality. On the other hand, a large proportion of multiple myeloma patients experience a prolonged asymptomatic period, and a small (but still significant) proportion are asymptomatic at diagnosis [1,2,3]. There is a need to fully elucidate the pathophysiological mechanisms involved in the development and progression of multiple myeloma, thereby facilitating the development of new therapeutic approaches based on these mechanisms and creating means to identify biomarkers that will enable early diagnosis or predict those with a poor prognosis. Oxidative stress occurs as a result of excessive production of ROS or insufficient elimination of ROS. Increased intracellular ROS production and/or decreased antioxidant response are established to facilitate malignant transformation, often due to oncogene activation and/or increased metabolism in tumor cells [5,7]. Although the possible pathophysiological effect of oxidative stress has been demonstrated in various types of cancer, including multiple myeloma, data on the relationship between multiple myeloma and oxidative stress are quite limited, especially in the context of determining which oxidative stress markers might be more involved in the diagnosis or prognostication of multiple myeloma [6,7,8]. In the present study, MDA and NO levels were found to be higher, and GSH-Px, SOD, and CAT levels were found to be lower in newly diagnosed multiple myeloma patients than in controls. In regression analysis, high MDA and NO levels, low GSH-Px, CAT, and SOD levels were identified as independent risk factors associated with multiple myeloma. In the ROC analysis, high classification properties were reported for MDA (>4.35), NO (>38.5), GSH-Px (<59.8), CAT (<67.2), and SOD (<21.2). However, the variables with the highest sensitivity and specificity were MDA and NO.

MDA is a reactive aldehyde formed as a byproduct of lipid peroxidation, which occurs when free radicals interact with polyunsaturated fatty acids in cell membranes. An increase in free radicals leads to overproduction of MDA [19,29,30,31]. It has been shown that MDA is highly reactive and mutagenic towards deoxyribonucleic acid [30]. NO is a signaling molecule that has various physiological effects, including vasodilatation, immune regulation, and pro- and anti-tumorigenic effects. Although it is important for normal cellular functions, under certain conditions, it reacts with ROS to form highly reactive nitrogen species, inducing oxidative stress. NO also facilitates angiogenesis, thereby potentially increasing blood and nutrient supply to cancer tissues. It can also inhibit apoptosis (programmed cell death), leading to tumor growth [32,33,34]. As mentioned previously, to counteract the harmful effects of oxidative stress, the body employs a complex network of defense mechanisms. The antioxidant system includes enzymes such as SOD, CAT, and GSH-Px, as well as non-enzymatic antioxidants such as glutathione, vitamins C, E, and A, and bilirubin [18,35]. SOD is an enzyme that plays an important role in detoxification by catalyzing the conversion of superoxide radicals to hydrogen peroxide and oxygen. Superoxide anions are the byproduct of various metabolic processes, including mitochondrial respiration, as well as the targeted product of specialized signaling enzymes. By means of their activity, SOD enzymes control the levels of various ROS and reactive nitrogen species, hence both limiting the potential toxicity and impacting a broad number of cellular functions, which are normally controlled through signaling functions. Changes in SOD expression affect cell survival and proliferation through ROS regulation [36,37]. CAT is an enzyme that integrates the antioxidant defense system and breaks down hydrogen peroxide into water and oxygen. It is abundant in almost all eukaryotic cells, especially in the peroxisomes and cytoplasm. It counteracts the toxic effects of hydrogen peroxide, which can cause cellular damage, thereby protecting the cell from oxidative stress. CAT is known as the first line of antioxidant defense, and it plays a critical role in preventing the formation of ROS as well as eliminating these compounds. To put it briefly, the literature demonstrates that CAT rapidly inactivates substances with potentially harmful effects on cells. Therefore, the determination of CAT activity is used as a useful and feasible biomarker for the evaluation of oxidation–reduction status. Moreover, alterations in CAT levels may impact the oxidative stress levels within cancer cells [31,38,39]. The glutathione system is one of the main antioxidant systems in cells and consists of glutathione, GSH-Px, and glutathione s-transferases. These systems reduce hydrogen peroxides or hydroperoxides using glutathione as a substrate and thus contribute to ROS detoxification [40]. The GSH-Px family is well known to perform many cellular functions, such as protection against oxidative damage, control of cellular processes such as apoptosis, growth and signaling, modulation of intracellular hydrogen peroxide levels, and maintaining overall intracellular redox balance. It is a very important antioxidant factor that has been associated with the prevention of chronic and degenerative diseases. Decreased levels of GPx-1 may result in the initiation of carcinogenesis, and in later stages of cancer, GSH-Px deficiency may even cause proliferative effects. However, adequate GPx-1 levels may block cell death through apoptosis, prevent deoxyribonucleic acid oxidation, help reduce the inflammatory process and thus contribute to increasing the chances of survival of transformed cells [31,40].

Numerous studies have demonstrated alterations in the levels of oxidant and antioxidant products in the context of cancer, including multiple myeloma [41,42,43]. Sharma et al. reported that multiple myeloma patients exhibited significantly lower levels of antioxidants such as SOD, GSH-Px, and CAT, as well as vitamins C and E, while their MDA levels were notably higher when compared to healthy controls [21]. Zima et al. found reductions in SOD and GSH-Px levels among multiple myeloma patients compared to controls. Interestingly, they did not observe a significant correlation between different disease stages and the concentrations of MDA or the activities of SOD and GSH-Px [27]. Another study, although without a control group, showed that SOD, GSH-Px, and CAT levels, as well as NO and MDA levels, were significantly lower in multiple myeloma patients treated with vincristine, adriamycin, and dexamethasone [24]. Gangemi and colleagues investigated oxidative stress parameters in untreated multiple myeloma patients and patients affected by monoclonal gammopathy of undetermined significance (MGUS). Comparisons with healthy controls have revealed that analysis of these parameters in multiple myeloma patients revealed a substantial increase in levels of AOPP, MDA, adenosine deaminase (ADA), and a marked decrease in total antioxidant capacity (TAC), glutathione, vitamins C and E, as well as antioxidant enzymes in multiple myeloma patients –both before and after treatment [23]. Furthermore, the authors performed longitudinal evaluations and showed that treatment resulted in significant increases in TAC, glutathione, vitamins C and E, and antioxidant enzymes. These effects were mirrored by the decreases in AOPP, MDA, and ADA levels [23]. In a prospective study, blood MDA levels were found to be higher, whereas SOD, CAT, glutathione, and glutathione-S-transferase activity levels were lower in multiple myeloma patients compared to control subjects. Furthermore, this study revealed that increased MDA and decreased SOD, CAT, and glutathione were directly associated with the stage of the disease [25], indicating that oxidative status (oxidant vs. antioxidant balance) could also be associated with prognosis or disease progression. Similar results indicating an imbalance of oxidative processes have been described in less comprehensive studies. For instance, Lodh et al. showed that MDA increased, and SOD levels decreased in patients with multiple myeloma compared to healthy controls [44]. Notably, such findings have been reported for quite some time, with older studies showing increased pro-oxidative markers and lower antioxidant markers in patients with multiple myeloma [26,27].

In both the present study and previous research, a consistent pattern emerges: oxidant levels tend to rise in multiple myeloma patients while the activities of antioxidant systems decrease. The precise cause-and-effect relationship between these two opposing systems and their roles in the pathogenesis of multiple myeloma remains unclear. Nevertheless, these collective findings strongly suggest a significant connection between oxidative stress and multiple myeloma, which is highly likely to be similar to the pathophysiological roles of ROS in other cancer types. A deeper understanding of these relationships could potentially lead to the development of innovative therapeutic approaches aimed at enhancing multiple myeloma treatment and ultimately improving patient outcomes. Consequently, a combined approach involving antioxidants or agents that modulate ROS may be considered to augment the efficacy of conventional cancer treatments such as chemotherapy and radiation. Furthermore, conducting more extensive studies is a must, especially since a large body of evidence on this topic is based on poor evidence and remarkably old studies. Our results identify MDA and NO as markers with high sensitivity and specificity for multiple myeloma detection; however, it is obvious that these are not specific markers for multiple myeloma and are nonspecifically associated with the presence and impact of oxidative stress. Nonetheless, since these parameters are easy to measure and cost-efficient, it may be possible to utilize their quantification as supportive data for diagnosis, potentially offering alternative venues to traditional diagnostic methods and costly confirmatory diagnostic tests. Perhaps more importantly, using these parameters to assess treatment response (since literature data supports this relationship) would reduce the costs of management and follow-up, which are typically based upon expensive and/or time-consuming analyses. However, it is important to acknowledge that new studies confirming our findings are necessary before any attempts can be made to create management protocols based on oxidative stress parameters. On the other hand, a significant proportion of patients with multiple myeloma are diagnosed after a long asymptomatic period. Nearly all multiple myeloma cases develop from an asymptomatic pre-malignant stage known as MGUS, which is seen in more than 3% of people over 50 years of age and can progress to multiple myeloma or an associated cancer (at a rate of 1% per year). More than 50% of individuals diagnosed with MGUS have had the condition for more than 10 years prior to multiple myeloma diagnosis [45,46]. Another important pre-diagnostic form of multiple myeloma is smoldering multiple myeloma (SMM), which is known as a more advanced premalignant stage. SMM progresses to multiple myeloma at a rate of about 10% per year in the first 5 years after diagnosis, 3% per year in the next 5 years, and 1.5% per year thereafter [47,48]. Some studies have been published on the possible associations of both MGUS and SMM with oxidative stress [49,50]. This issue was not investigated in the present study but investigating the pathophysiological triggers of these two clinical entities, which are possible precursors of multiple myeloma, and elucidating the role of oxidative stress in the development of these conditions and their transformation into multiple myeloma may allow multiple myeloma to be detected at an asymptomatic stage. In addition, to establish a definitive pathophysiological bridge between multiple myeloma and oxidative stress, the relationship between multiple myeloma stages and oxidative stress markers must be investigated, and possible significant changes in the amounts of oxidative stress markers before and after treatment of multiple myeloma must be demonstrated. Finally, continued research is essential to unravel the complexities of these interactions and their validity in different populations and different disease stages.

When interpreting our results, it is crucial to consider certain limitations. This study requires replication with larger sample sizes and broader data collection, as the dataset examined and the study group are small to reach definitive conclusions. For instance, the lack of other laboratory results from participants, especially those that could influence oxidative stress (anemia, liver function tests, kidney function tests, parameters related to lipid and glucose metabolism, and blood pressure measurements), may impact the reliability of the regression analyses. However, if such data were to be included, the small population size would have become an insurmountable obstacle that would prevent multivariable regression. This study did not investigate the relationship between multiple myeloma stages and oxidative stress markers. A significant proportion of newly diagnosed multiple myeloma patients may be asymptomatic at the time of diagnosis. On the other hand, increased calcium levels can be used as an early indicator of multiple myeloma [51]. Increased ROS may target calcium channels, which may lead to calcium elevation and further increased ROS levels. Whether ROS levels are a more sensitive indicator for early diagnosis should be investigated in future comprehensive longitudinal studies that collect longitudinal calcium data. The fact that calcium levels were not compared with ROS levels in this study can be considered another limitation. Additionally, various other oxidative stress markers were not assessed, including total oxidant and antioxidant capacity, AOPP, vitamin C and E levels, ADA levels, and glutathione and glutathione-S-transferase activity levels. These limitations underscore the need for more comprehensive investigations that include larger patient groups and greater datasets to accurately assess the relationship between oxidative stress and multiple myeloma.

## 5. Conclusions

In our investigation into oxidative stress-related biomarkers among patients with multiple myeloma, our findings reveal a significant association between elevated levels of MDA (>4.35), NO (>38.5), decreased GSH-Px level (<59.8), diminished CAT level (<67.2), and reduced SOD level (<21.2) with multiple myeloma. Furthermore, classification data showed that MDA and NO were the most sensitive oxidative stress markers associated with the presence of multiple myeloma. Clarifying the pathophysiological relationship between multiple myeloma and oxidative stress and the utility of these biomarkers in multiple myeloma diagnosis and management may contribute to the development of new and more effective treatment agents for multiple myeloma and to easier and more effective management of multiple myeloma.

## Figures and Tables

**Table 1:** Summary of demographics and antioxidant levels with regard to groups.

	Groups		*p*
	Controls (*n* = 30)	Patients (*n* = 30)	
Age (years)	65.13 ± 8.80	63.87 ± 10.93	0.623
Sex			
Male	17 (56.67%)	18 (60.00%)	1.000
Female	13 (43.33%)	12 (40.00%)	
Comorbidities			
Diabetes mellitus	5 (16.67%)	7 (23.33%)	0.747
Hypertension	12 (40.00%)	11 (36.67%)	1.000
Ischemic heart disease	3 (10.00%)	7 (23.33%)	0.299
COPD	2 (6.67%)	3 (10.00%)	1.000
Hypothyroidism	1 (3.33%)	2 (6.67%)	1.000
MDA (nmol/mL)	3.80 (3.04–4.12)	8.49 (6.94–10.34)	<0.001
GSH-Px (ng/mL)	78.36 (60.42–85.12)	57.30 (50.73–72.04)	<0.001
CAT (ng/mL)	85.45 (69.95–93.73)	59.53 (55.52–71.05)	<0.001
SOD (ng/mL)	31.94 ± 9.80	22.89 ± 9.40	0.001
NO (µmol/L)	27.85 (13.04–36.49)	54.60 (49.46–64.89)	<0.001

Data are given as mean ± standard deviation or median (1st quartile–3rd quartile) for continuous variables according to the normality of distribution and as frequency (percentage) for categorical variables. Abbreviations; CAT: Catalase, COPD: Chronic obstructive pulmonary disease, GSH-Px: Glutathione peroxidase, MDA: Malondialdehyde, NO: Nitric oxide, SOD: Superoxide dismutase.

**Table 2:** Performance of antioxidants to predict multiple myeloma.

	Cut-Off	Sensitivity	Specificity	Accuracy	PPV	NPV	AUC (95% CI)	*p*
MDA (nmol/mL)	>4.35	96.67%	100.00%	98.33%	100.00%	96.77%	0.967 (0.902–1.000)	<0.001
GSH-Px (ng/mL)	<59.8	70.00%	90.00%	80.00%	87.50%	75.00%	0.773 (0.645–0.901)	<0.001
CAT (ng/mL)	<67.2	73.33%	86.67%	80.00%	84.62%	76.47%	0.850 (0.755–0.945)	<0.001
SOD (ng/mL)	<21.2	56.67%	86.67%	71.67%	80.95%	66.67%	0.760 (0.639–0.881)	0.001
NO (µmol/L)	>38.5	96.67%	90.00%	93.33%	90.63%	96.43%	0.960 (0.895–1.000)	<0.001

Abbreviations: AUC: Area under ROC curve, CAT: Catalase, CI: Confidence interval, GSH-Px: Glutathione peroxidase, MDA: Malondialdehyde, NO: Nitric oxide, NPV: Negative predictive value, PPV: Positive predictive value, SOD: Superoxide dismutase.

**Table 3:** Odd ratios for multiple myeloma and logistic regression analysis results.

	Unadjusted		Adjusted ^(1)^	
	OR (95% CI)	*p*	OR (95% CI)	*p*
MDA (nmol/mL)	5.679 (1.755–18.380)	0.004	6.336 (1.886–21.289)	0.003
GSH-Px (ng/mL)	0.937 (0.900–0.974)	0.001	0.931 (0.892–0.972)	0.001
CAT (ng/mL)	0.915 (0.874–0.958)	<0.001	0.910 (0.865–0.957)	<0.001
SOD (ng/mL)	0.898 (0.838–0.963)	0.003	0.895 (0.833–0.962)	0.003
NO (µmol/L)	1.217 (1.087–1.362)	0.001	1.217 (1.084–1.366)	0.001

Abbreviations: CAT: Catalase, CI: Confidence interval, GSH-Px: Glutathione peroxidase, MDA: Malondialdehyde, NO: Nitric oxide, OR: Odds ratio, SOD: Superoxide dismutase, (1) Adjusted using age, sex, and comorbidity.

## Data Availability

Data are available after a request to the corresponding author.
